# Early phase of COVID-19 quarantine impacted insomnia symptoms in Turkish families

**DOI:** 10.5935/1984-0063.20220019

**Published:** 2022

**Authors:** Huri Seval Gönderen Çakmak

**Affiliations:** Çankiri Karatekin University, Eldivan Vocational School of Health Services - Çankiri - Turkey.

**Keywords:** Insomnia, COVID-19, Sleep, Sleep Stages

## Abstract

Quarantine and isolation are the most important public health measures used to prevent the transmission of infectious diseases among individuals. Quarantine and social isolation cause some health problems, such as depressive symptoms, sleep disturbances, anxiety, irritability, feeling of loneliness, helplessness, and trauma and post-traumatic stress symptoms. In this study, we aimed to examine early phase of COVID-19 quarantine impacted insomnia symptoms in Turkish families. The population of the study comprised students attending the Department of Healthcare Services of Eldivan Health Vocational School at Çankırı Karatekin University and their families. In total, 564 students and their families completed online surveys. Data was collected with Google forms. Snowball sampling was used as the data collection method. Female participants, those who gained weight during home quarantine, those who put their phones under their pillow or at the bedside and in another room, and those who used a phone or tablet before sleeping were found to have significantly more insomnia compared to others. The mean day of uninterrupted home quarantine was higher for participants experiencing insomnia (p<0.05). This study showed that individuals experienced sleep problems during the home quarantine.

## INTRODUCTION

After the World Health Organization (WHO) declared COVID-19 to be a pandemic, schools, restaurants, entertainment centers, shopping malls, factories, and hairdressers were closed in most countries to prevent spread of the virus. In Turkey, young people between 0-20 years and older adults over 65 years were subjected to restrictions about leaving home after the decision of the Ministry of Health Pandemic Science Board. People between the ages of 20-65 can only go out for a short time in compulsory situations (market, emergency situations, etc.). Curfews were declared in some of the big cities where a significant number of cases were reported. Quarantine and isolation are the most important public health measures used to prevent the transmission of infectious diseases among individuals. Although people had to stay more in their homes and their living spaces were restricted, COVID-19 positive cases decreased thanks to these measures^1,2^. In addition, the quarantine process digitalized people’s social activities and habits, changing both melatonin mechanism and sleep rhythm^3^.

Recent studies found that quarantine and social isolation cause some health problems, such as depressive symptoms, sleep disturbances, anxiety, irritability, feelings of loneliness, helplessness, and trauma and post-traumatic stress symptoms^1,2,4,5,6,7^. A study conducted in China during the COVID-19 pandemic also found that about 35% of those surveyed showed psychological distress^8^. In a study conducted with 129 people with severe acute respiratory syndrome in Canada, those who were quarantined for more than 10 days showed significantly higher post-traumatic stress symptoms than those who were quarantined for less than 10 days. In addition, 28.9% of individuals had symptoms of post-traumatic stress disorder and 31.2% had symptoms of depression^9^. In a study conducted by Shi et al. during the pandemic in China, the rates of mental health symptoms among the respondents during the COVID-19 pandemic were found to be 27.9% for depression, 31.6% for anxiety, 29.2% for insomnia and 24.4% for acute stress^10^.

It should be noted that sleep is an essential determinant of the general health status of an individual, as well as contributing to the functioning of the defense mechanism against infections. Individuals with quarantine or limited social relationships are known to experience many health problems related to insomnia ^11,12,13^.

Impairment of sleep quality not only ruins the general health status of an individual but also makes it difficult to cope with the COVID-19 infection due to changing the immune response^14^. There is solid evidence to suggest the role of sleep in strengthening immunity^15^. In the study by Hajak and Group (2001), insomnia patients were found to visit hospital, to suffer tics, and to be medicated more than their healthy counterparts. In addition, insomnia adversely affects patients’ quality of life^16^.

Sleep plays a fundamental role in mental and physical health. Adequate sleep time and quality are essential to cope with major vital events such as the COVID-19 pandemic^17^. Stress sleep problems are common, and people with stress-related sleep disorder have a higher risk of developing chronic insomnia^12^. In a study by Kocevska et al., 20% of individuals with good sleep quality simultaneously had seriously decreased sleep quality after quarantine. Changes in sleep quality throughout the pandemic were associated with negative affect and anxiety^18^. Similarly, in a study conducted in France, sleep problems were observed in individuals during the closures due to COVID-19^19^. A study in China also found that low sleep quality was quite common in individuals returning to work during COVID-19^20^. A study conducted in Norway found that anxiety and depressive symptoms were associated with sleep disorders^21^. Insomnia is generally associated with depression, anxiety, and mental disorders^22^.

Although the world and Turkey initiated the normalization process from June 2020, it is prudent to say that people have not fully restored their habits from before the pandemic, such as spending time in shopping malls and playgrounds and going to the cinema. The relevant authorities, such as the World Health Organization, state that COVID-19 outbreak measures may continue for 1 to 2 years, which means that a chronic process awaits society after the acute process. Quarantine is generally an unpleasant experience. Separation from beloved ones, restrictions on freedom, uncertainty about illness, and boredom can have dramatic effects on sleep quality. For this reason, health professionals, who lead the way in maintaining and supporting public and individual health, should evaluate the situation and develop alternative interventions that can be applied at home both for the protection of the general health status of individuals and for effective interventions against COVID-19.

In this study, the aim was to determine early phase of COVID-19 quarantine impacted insomnia symptoms in Turkish families.

## MATERIAL AND METHODS

### Study design and Participants

This study was conducted as a descriptive cross-sectional study. The population of the study included students attending the Department of Healthcare Services of Eldivan Health Vocational School at Çankırı Karatekin University and their families. The study was collected by Google forms due to pandemic conditions. The study link was sent to the mobile phones of students through the health care services department. Those who accepted participation in the study were asked to fill in the study link and then send it to their families. In this way, the snowball sampling method was applied.

A total of 250 students were enrolled in the department, but the population was calculated as 750 people, considering that students would send study links to at least 3 family members. The inclusion criteria were being 18 years of age and over and accepting participation in the study. Overall, 564 people (75% of the universe) participated in the study.

### Data collection

Due to the COVID-19 outbreak, the data were gathered through online survey forms between June 08-30, 2020. The survey link was sent to the students and their family members via SMS, and they were asked to fill in the survey forms. Participation in the study was on a totally voluntary basis.

### Data Collection Tools

#### Demographic Information Form

Considering the previous research, a demographic information form was created by the researcher to identify the socio-demographic characteristics of the participants^3,14,23,24,25,26,27^. This form contained 11 questions related to age, gender, weight, educational attainment, use of phone or tablet, and smoking.

### Bergen Insomnia Scale

The scale was developed by Pallesen et al. in 2008 based on official and clinical diagnostic criteria for insomnia. The Bergen Insomnia Scale was created on the basis of DSM-4 diagnostic criteria, but it also meets the DSM-5 diagnostic criteria. The scale consists of six questions measuring different symptoms of insomnia. Using the 8-point Likert-type scale, respondents express the number of days that they experienced sleep problems over the past month. While the lowest score that can be obtained from the scale is 0, the highest score is 42. Higher scores indicate more sleep and daytime fatigue. Getting a score of 3 or more from at least one of the first three questions of the scale and additionally getting a score of three or more from at least one of the last two questions can be stated as insomnia. Pallesen et al. (2008) administered the scale to three different sample groups: students, communities, and patients. While a two-factor structure was found in the student and patient samples, the scale showed a single-factor structure in the community sample. In the original study conducted with adults, the internal consistency coefficient of the scale was found to be α = 0.87, and its test-retest reliability coefficient was calculated as r = 0.77. According to DSM-5 diagnostic criteria, 3 or more points from at least one of the first three questions or one of the last two questions indicate insomnia. In recent studies, the fourth question on the scale, “*How many days a week do you feel unable to rest enough after waking up*” was indicated to be related to sleep quality; therefore, it should be evaluated within DSM-5 criteria^28^.

The validity and reliability studies of the Turkish version of the Bergen Insomnia Scale were performed by Bay, Yalçın, and Ergün (2018). A two-factor structure was found in the Turkish version of the scale administered to adult individuals. Cronbach’s Alpha reliability coefficient was calculated as 0.85 for the daytime symptoms sub-scale, 0.80 for the nighttime symptoms sub-scale and 0.85 for the total scale^29^.

### Statistical analysis

The data about demografic variables are presented as numbers and percentages. Fisher’s Exact Test, Pearson’s Chi Square Test, and Yates Correction were used for categorical variables. The Shapiro-Wilk test was used to check the normality of continuous variables, and the not normally distributed data were analyzed with the Mann-Whitney U Test (nonparametric test). The statistical significance level was accepted as p <0.05.

## RESULTS

The mean age of the participants was 28.13 ± 0.49 years (min: 18 / max: 90). While 71.1% of the participants were female, 66.5% were single. It was found that 66.3% of the individuals were university students, and 82.8% did not have any chronic diseases. Twenty-five percent of the participants lived alone, 32.4% lived with their parents, and 41.7% lived with their children and spouses. While 72.0% did not smoke, 42.9% of the participants stated that they gained weight during quarantine. Sixty-four percent stated that they kept their phone under their pillow/at the bedside, while 83.3% stated that they used a tablet/phone before sleeping. The results revealed that 64.7% of the participants had insomnia (Table 1).

**Table 1:** Socio-demographic characteristics and insomnia findings of the participants.

Variable	Number (n:564)	%
**Age**	**28.13 ±0.487 (min:18/max:90)**	
**Gender**		
Female	401	71.1
Male	163	28.9
**Marital status**		
Single	375	66.5
Married	189	33.5
**Educational attainment**		
Literate	6	1.1
Primary school graduate	46	8.2
High school graduate	88	15.6
University student	374	66.3
University graduate	50	8.9
**Chronic disease**		
Yes	97	17.2
No	467	82.8
**Who do you live with at home?**		
Mother - father	183	32.4
Children-spouse	235	41.7
Alone	146	25.9
**Smoking status**		
Yes	144	25.5
No	406	72.0
I haven′t been smoking for the past 6 months	14	2.5
**Smoking during quarantine? n: 144**		
Increased	28	19.44
Decreased	22	15.27
Unchanged	95	65.97
**Weight Status During Quarantine**		
Increased	242	42.9
Decreased	113	20.0
Unchanged	209	37.1
**Where do you put your phone/tablet while sleeping?**		
Under my pillow / at my bedside	361	64.0
Away from my bed	157	27.8
In another room	46	8.2
**Do you use a phone/tablet before going to sleep?**		
Yes	470	83.3
No	94	16.7
**Insomnia fnding**		
Insomnia	365	64.7
No insomnia	199	35.3

The mean age of the participants was 28.13 ± 11.56 years, the mean days of uninterrupted home quarantine was 34.42 ± 31.13, and the mean scores of the participants on the scale was 18.26 ± 10.29 (Table 2). The findings suggest that there was no statistically significant difference between insomnia and age, educational attainment, smoking status, and who the participants lived during the quarantine (p>0.05). In addition, female participants (p = 0.001), those who gained weight during the home quarantine (p = 0.031), those who put their phones under the pillow or at the bedside (p = 0.016) and in another room (p = 0.016), and those who used a phone or tablet before sleeping (p = 0.020) were found to have significantly higher levels of insomnia than others. According to the findings on the scale, there was a statistically significant difference between interrupted quarantine and uninterrupted quarantine (**p: 0.012**) (Table 3). The mean days of uninterrupted home quarantine was higher for participants experiencing insomnia.

**Table 2:** Mean, minimum, and maximum values for age, uninterrupted home quarantine, and the scale.

	M ±SD	Min.	Max.
**Age**	28.13 ± 11.563	18	90
**Home quarantine**	34.42 ±31.127 (days)	1	120
**Scale score**	18.26 ± 10.288	0	42

**Table 3:** Insomnia according to socio-demographic characteristics of the participants.

		Bergen Insomnia Scale				
Variable						
		Insomnian	%	No insomnian	%	Statistics
**Age groups**						
	18-24 years	220	68.1%	103	31.9%	χ2 : 5.627*
	25-34 years	57	56.4%	44	43.6%	
	35-44 years	49	60.5%	32	39.5%	p=0.344
	45-55 years	25	64.1%	14	35.9%	
	56-64 years	8	72.7%	3	27.3%	
	65 years and over	6	66.7%	3	33.3%	
**Gender**						
	Female	277	69.1%	124	30.9%	χ2:11.556**
	Male	88	54.0%	75	46.0%	p=0.001
	Literate	5	83.3%	1	16.7%	χ2:8.112*
**Educational Attainment**						
	Primary school graduate	31	67.4%	15	32.6%	p=0.088
	High school graduate	48	54.5%	40	45.5%	
	University student	253	67.6%	121	32.4%	
	University graduate	28	56.0%	22	44.0%	
**Who do you live with at home?**						
	Mother - father	123	67.2%	60	32.8%	χ2:0.817**
	Children-spouse	148	63.0%	87	37.0%	
	Alone	94	64.4%	52	35.6%	p=0.665
**Smoking status**						
	Yes	87	60.4%	57	39.6%	χ2:1.589*
	No	269	66.3%	137	33.7%	
	I haven′t been smoking for the past 6 months	9	64.3%	5	35.7%	P=0.452
**Smoking during quarantine? n: 144**						
	Increased	22	78.57	6	21.42	χ2:6.790**
	Decreased	14	63.63	8	36.36	p=0.147
	Unchanged	52	55.31	42	44.68	
**Weight Status During Quarantine**						
	Increased	168	69.4%	74	30.6%	χ2:6.925***
	Decreased	76	67.3%	37	32.7%	
	Unchanged	121	57.9%	88	42.1%	p=0.031
**Where do you put your phone/tablet while sleeping?**						
	Under my pillow / at my bedside	31	67.4%	15	32.6%	χ2:8.264***
	Away from my bed	247	68.4%	114	31.6%	p=0.016
	In another room	87	55.4%	70	44.6%	
**Do you use a phone/ tablet before going to sleep?**	Yes	315	67%	155	33%	χ2:7.814*
	No	50	53.8%	43	46.2%	p=0.02
**Uninterrupted quarantine time (days)**		M	SD	M	SD	Z: −2.512
		34.48	31.66	30.29	29.72	p: 0.012

*Fisher’s Exact Test,

** Pearson’s Chi Square Test,

***Yates Correction, Z= Mann-Whitney U Test.

## DISCUSSION

This study, using data collected from Turkish individuals during the COVID-19 pandemic, aimed to determine early phase of COVID-19 quarantine impacted insomnia symptoms in Turkish families.

In the current study, 64.7% of the individuals participating in the study experienced insomnia according to the Bergen insomnia scale. Similarly, in a study conducted with university students, it was determined that 59.3% of the students experienced insomnia^30^. In a study conducted in the USA, approximately 35% of Americans complained of insomnia at least once in a year^31^. During the pandemic period, insomnia was reported in 37.6% of the Greek population^32^. In Italy, 57% of individuals reported low sleep quality during the pandemic^33^. The reason for this difference may be the cultural structure and the home quarantine processes experienced during the pandemic differ from country to country.

In the current study female participants were found to suffer more insomnia than males (**p=0.001**). The literature suggests that insomnia is seen more frequently in females, the divorced, the unemployed, older adults, and individuals with poor educational background and low socioeconomic status^24,34,35^. In the study by Parlapani et al., women reported significantly higher levels of fear associated with COVID-19, more severe depressive symptoms, sleep disturbances, and higher levels of intolerance to uncertainty^36^. We can attribute the higher incidence of insomnia in women to causes such as the perception of stress and inactivity ^30^. Also, previous research has found that people with depression and anxiety are very likely to experience insomnia. The fact that the female gender is more prone to depression and anxiety contributes to the possibility of insomnia^37,38^.

In our study, the reason for insomnia in more than half of the participants can be explained by the home quarantine due to the COVID-19 outbreak. A study suggested that the more time adults spent watching TV and using the internet before sleeping, the more likely they were to report poor sleep^39^. In another study, the use of a computer or mobile phone in bedroom was associated with poor sleeping habits, but the use of digital tools in the bedroom was unrelated to signs of insomnia^40^. In the current study, it was observed that those who put their phones or tablets under the pillow or at the bedside and those using a phone or tablet before sleeping experienced more insomnia than others. Demirer et al. found the rate of insomnia was significantly higher for those who use phones in bed than those who do not, and using a phone in bed increased the risk of insomnia 1.6 times ^30^.

Social isolation during the pandemic has undoubtedly increased people’s habits of using digital tools. This increase also affects secretion of melatonin due to factors such as exposure to light at night and late bedtimes. Melatonin can reduce immune suppression caused by chronic stress and sleep deprivation ^41^.

In China, insomnia in the general population was assessed at 15%, and the widespread use of new media such as computers and smartphones in young adults was associated with an increased risk of insomnia^42^. The relationship between smoking and sleep disturbance is well known. Smoking is associated with shorter sleep time and increased difficulty falling asleep, as well as other sleep disorders^26,43^. Nevertheless, there was no statistically significant difference between smokers and non-smokers regarding insomnia (p=0.452), which was also the case for those who increased smoking during the home quarantine and those who did not (p=0.147). In the current study, those who gained weight during the home quarantine had more impaired sleep than others (**p=0.031**). Weight increase was observed in 42.9% of the participants. Zachary et al. (2020) in a study found roughly 22% of adults reported gaining weight during the COVID-19 outbreak. During this period, eating due to lack of sleep, decreased physical activity, and eating in response to stress are behaviors related to weight gain during quarantine. Just as a lack of sleep increases eating, weight gain also causes sleep problems.

Suffering from insomnia increased significantly as the duration of uninterrupted quarantine increased (**p= 0.012**). Although the duration of quarantine is not always definite, a study showed that those who were quarantined for more than 10 days showed significantly higher post-traumatic stress symptoms than those quarantined for less than 10 days^9^. Some studies showed that longer quarantine times were associated with poor mental health, especially with post-traumatic stress symptoms ^44,45^. Therefore, prolongation of the quarantine time may affect habits, eating behaviors, and internet and substance use. It was shown in a study that isolated individuals had high levels of anxiety and stress and low sleep quality. However, being relaxed and sleeping well are all required for good psychological health of individuals isolated during the epidemic. Additionally, social capital levels can affect mental health and sleep quality. Social capital includes social trust, belonging, and social participation. During 14-day self-isolation due to the COVID-19 outbreak in central China, increased social capital improved sleep quality by reducing anxiety and stress^27^. In addition, social interactions can reduce negative emotions and improve mood. It was concluded in a study that social support for healthcare staff positively affected their sleep quality.^46^.

Although sleep problems due to quarantine during COVID-19 in France began to return to normal after closure, improvements in sleep problems decreased especially in those exposed to negative news about the epidemic^19^. In a study conducted in Spain, 39.7% of individuals reported lower quality sleep during COVID-19 and this had a positive correlation with BMI^47^.

## LIMITATIONS

The current study had several limitations. Firstly, insomnia symptoms were evaluated according to the self-report of individuals. Secondly, we do not know the anxiety and depression levels of individuals before the pandemic. If individuals have pre-pandemic anxiety and depression, they may be more likely to experience insomnia symptoms.

## STRENGTHS

Moreover, participants experience of insomnia was determined only based on their statements. This study is the first nursing study to evaluate early phase of COVID-19 quarantine impacted insomnia symptoms in Turkish families. This study can serve as a basis for evaluating sleepiness and sleep habits of individuals during and after the quarantine period.

## CONCLUSION

To the best of our knowledge, this is the first study investigated early phase of COVID-19 quarantine impacted insomnia symptoms in Turkey. This study showed that individuals experienced sleep problems during the home quarantine. Therefore, the results of the study form a robust ground for nursing interventions so that individuals can maintain healthy habits in spite of sudden lifestyle changes during a pandemic. During the pandemic, individuals can be followed up with tele-nursing practices so that they can maintain their healthy sleep habits. In addition, individuals can be directed to detox for internet and computer use.
